# Aptamer-Based Gold Nanoparticles–PDMS Composite Stamps as a Platform for Micro-Contact Printing

**DOI:** 10.3390/bios12121067

**Published:** 2022-11-23

**Authors:** Amna Didar Abbasi, Zakir Hussain, Kun-Lin Yang

**Affiliations:** 1 Department of Materials Engineering, School of Chemical and Materials Engineering (SCME), National University of Sciences and Technology (NUST), Islamabad 44000, Pakistan; 2 Department of Chemical and Biomolecular Engineering, National University of Singapore, Engineering Drive 4, Singapore 117576, Singapore

**Keywords:** liquid crystals, DMOAP, AuNPs–PDMS composite, micro-contact printing

## Abstract

In the present study, a functional template made up of in situ synthesised gold nanoparticles (AuNPs) is prepared on polydimethylsiloxane (PDMS) for patterning of target protein onto the desired solid substrates. Unlike previous studies in which bioreceptor probes are randomly attached to the PDMS stamp through electrostatic interactions, herein, we propose an AuNPs–PDMS stamp, which provides a surface for the attachment of thiol-modified biorecognition probes to link to the stamp surface through a dative bond with a single anchoring point based on thiol chemistry. By using this platform, we have developed the ability for microcontact printing (µCP) to selectively capture and transfer target protein onto solid surfaces for detection purposes. After µCP, we also investigated whether liquid crystals (LCs) could be used as a label-free approach for identifying transfer protein. Our reported approach provides promise for biosensing of various analytes.

## 1. Introduction

Patterning of biomolecules finds many applications in biomedical research, including biosensing, diagnostic immunoassays, DNA hybridisation and many others [1,2,3,4]. In biosensing, the most crucial step of sensor construction is the active placement of the biorecognition element on to the substrate. For active placement of biorecognition elements on surfaces, many procedures such as inkjet printing, micro arraying, electrospray and patterning through AFM tip demonstrate great promise. However, these procedures result in unclear patterns, protein aggregation and loss of biological activity [5]. In contrast, μCP is an efficient and simple procedure for direct patterning biorecognition elements (proteins, antibodies and oligos) on solid substrates without the need for photolithographic tools and a clean room facility [6,7].

Moreover, proteins cannot be synthesised on solid substrates but can be patterned. However, some proteins may not survive adsorption processes on surfaces. For these sensitive proteins, μCP is an ideal patterning option. In addition, μCP combines well with many biomedical procedures such as enzyme-linked immunosorbent assay (ELISA), microfluidic networks and fluorescence labelling [6].

The excellent properties exhibited by PDMS, including biocompatibility, bio- and oxidative stability, non-toxicity, optical transparency, flexibility, simple fabrication and good mechanical properties, make PDMS an ideal candidate for use as a stamp in μCP [8,9]. Despite these remarkable properties, the hydrophobicity of PDMS (water contact angle ~108° ± 7°) often limits its application in sensor construction where the active placement of biomolecules is concerned. The undesired non-specific adsorption of proteins due to the hydrophobic nature of the PDMS surface results in compromised detection sensitivity [8,9,10]. 

Recently, in order to introduce biomolecules on the inert surface of PDMS, metal nanoparticle incorporation, especially AuNPs in a polymer matrix, has gained much interest in various fields, including in biosensing and optical devices [10,11,12,13,14,15,16,17]. As reported in earlier studies, thiol-containing molecules may directly interact with metal surfaces to form dative bonds [18]. Zhang et al. have proposed a simple in situ fabrication method to prepare an AuNPs–PDMS composite without the need for additional reducing or stabilising agents [12]. Since the inclusion of AuNPs on the PDMS surface makes it a suitable platform for attachment of biorecognition probes (aptamer, proteins or antibodies) with thiol modification [11,14,19], we propose this composite as a stamp material for attachment of a thiol-modified aptamer based on gold-thiol chemistry, which selectively binds with its target protein, and then transferring of target protein on the desired substrate through μCP. At the same time, aptamers are synthetic single-stranded oligonucleotides (RNA or DNA) that have been exploited as biorecognition probes in various bioassays due to their high specificity and selectivity [20,21,22,23]. In this study, we chose the B40t77 RNA aptamer as a biorecognition probe for HIV-1 surface glycoprotein gp-120. Initially, it was created for HIV-1 therapeutics [24]. We have recently used the B40t77 aptamer to develop bioassays for gp-120 [21,23]. The key steps involved in μCP are: (1) fabrication of PDMS stamps; (2) in situ synthesis of gold nanoparticles on the surface of an as-prepared PDMS stamp; (3) linking the stamp with a thiol-modified aptamer and incubating these stamps with the target protein before μCP; and (4) conformal contact of aptamer-conjugated AuNPs–PDMS stamps with the UV treated DMOAP-coated glass slide. In addition, liquid crystals (LCs) have recently drawn much attention due to their distinct optical characteristics and long range orientational order. Many researchers have exploited LCs as a signal transducer for label-free detection of biomolecules [25,26,27,28,29,30]. To analyse the fate of transferred protein, after μCP we utilized the LCs orientational response towards patterned protein on a DMOAP-coated slide, which yields optical readouts that are visible to the naked eye under crossed polarizers.

In the present study, we demonstrated a AuNPs–PDMS stamp as a stable platform for the attachment of a thiol-modified biorecognition probe based on thiol chemistry. We used a thiol-modified RNA aptamer (B40t77 aptamer) as a probe to check the interaction between probe oligos and stamp. Furthermore, the gp-120 protein was used as a target protein. Before conformal contact with the desired substrate, the aptamer bound AuNPs–PDMS stamp was incubated with a solution containing gp-120 protein. Then the PDMS stamp with the B40t77aptamer-gp-120 complex was conformally contacted with a UV exposed DMOAP-coated glass slide to transfer gp-120 to the DMOAP-coated glass substrate. This glass substrate with transferred protein was then used to fabricate LCs optical cells and analysed under the polarised optical microscope for results. Furthermore, by using 3′ 6 FAM-conjugated thiolated Apt 8 aptamer, we further confirmed that AuNPs–PDMS stamps are ideal stamp material for attachment of thiol-modified biorecognition probes. 

## 2. Materials and Methods

### 2.1. Materials

PDMS monomer and curing agent (Sylgard 184) was purchased from Dow Corning (Midland, MI, USA). Glycoprotein-120 was obtained from Abcam (Cambridge, UK). FITC-hIgG was procured from Sigma Aldrich (Singapore). 2-fluoro pyrimidine substituted 77 nucleotides long B40t77 RNA aptamer was custom synthesised by Gene Link (Hawthorne, NY, USA). 2-fluoro pyrimidine substituted 23 nucleotides long Apt 8 RNA aptamer with 3′ 6-FAM and 5′ thiol modification was custom synthesised by IDT (Singapore). Tris EDTA (1× T.E., pH-8) and PBS buffer (10×, pH-7.4) were obtained from 1st BASE (Singapore). Decon-90 was purchased from VWR (Singapore). Microscopic glass slides were purchased from Merienfield (Berlin, Germany). *N*,*N*-dimethyl-*n*-octadecyl-3aminopropyltrimethoxy-silyl chloride (DMOAP), RNase-free water and magnesium chloride were purchased from Sigma Aldrich (Singapore). Liquid crystals 4-cyano-4-pentylbiphenyl (5CB) were obtained from Merck (Tokyo, Japan). Mylar transparent film was procured from Infinite Graphics (Singapore). Gp-120 stock and working solutions were prepared in 0.01 M phosphate-buffered saline (PBS, pH-7.4). Aptamer stock solutions were prepared by dissolving B40t77 and Apt 8. The stock solutions were prepared in RNase-free water at room temperature and further dilutions were prepared in T.E. buffer with 100 mM of magnesium chloride. To ensure correct spatial folding, the aptamer solution was first heated to 95 °C for 3 min, followed by cooling at room temperature for 5 min, and lastly, the solution was cooled on ice. 0.2 µm filtered Milli-Q water (Milli-Q system, Billerica, MA, USA) was utilized to prepare all buffer solutions.

### 2.2. Glass Slides Cleaning and Surface Modification with DMOAP

Glass slides were twice cleaned with deionized water followed by overnight soaking in a solution containing 5 percent (*v*/*v*) Decon-90. Next, the glass slides were ultrasonically cleaned for 15 min with deionized water before being thoroughly rinsed with deionized water. For surface modification of glass slides with DMOAP, the cleaned glass slides were immersed for 5 min in a solution containing 0.1 percent (*v*/*v*) DMOAP. The glass slides were then rinsed with deionized water 5 times to eliminate remaining unreacted DMOAP from the surface and afterwards dried under a compressed stream of nitrogen gas. Lastly, to crosslink the immobilised DMOAP, these glass slides were heated to 100 °C in a vacuum oven for 15 min.

### 2.3. Preparation of PDMS Stamps

To prepare the PDMS stamp, the prepolymer mixture (silicon elastomer and curing agent) was first mixed thoroughly in a weight ratio of 10:1. After mixing, the air bubbles were removed by degassing the mixture in a vacuum desiccator for 1.5 h after pouring it on a cleaned Petri plate. After that, the prepolymer mixture was cured at 65 °C for 6–7 h. Finally, the cured PDMS was carefully peeled off the Petri plate and cut into the appropriate size. To clean the PDMS, it was soaked in absolute ethanol overnight, washed with deionised water and dried under a stream of nitrogen. Finally, the cleaned PDMS stamp was baked at 65 °C for 60 min to vaporise any ethanol trapped inside it.

### 2.4. U.V. Treatment of PDMS Stamp and DMOAP-Coated Slide

Before modification, the surface of the prepared PDMS stamp and DMOAP-coated glass slide was placed under a UV pen lamp (254 nm, model 11SC-1, Sigma-Aldrich, St. Louis, MO, USA) for durations of 5 and 1 min, respectively. The distance between the surface of the PDMS stamp and DMOAP-coated glass slide, and the UV pen lamp was kept constant at 1.5 cm throughout all experiments. 

### 2.5. Preparation of AuNPs–PDMS Composite Stamp

PDMS–AuNPs composite film was prepared according to the procedure in the literature [12,19]. Briefly, prepared PDMS pieces were incubated with drops of 15 µL of 0.5% HAuCl_4_·3H_2_O solution in the form of the array at 37 °C in a self-made humidified chamber for 48 h. The PDMS pieces were then thoroughly rinsed with 0.22 µm filtered milli-Q water, followed by drying under a stream of nitrogen gas. They were then stored at 4 °C in the refrigerator for further use.

### 2.6. Immobilisation of B40t77 Aptamer on AuNPs–PDMS Stamp 

Drops of 15 µL pretreated aptamer solution were dispensed on the cleaned and dried piece of PDMS–AuNPs composite stamp in the form of a small array with the help of a micropipette and then kept in a self-made humidified chamber at 4 °C for 12 h. It was reported previously that the kinetics of modification of AuNPs surfaces with thiols could take more than one hour to complete [18]. After incubation, they were thoroughly rinsed with RNAase-free water to remove any unbound or physically bound aptamer from the surface and then dried under a nitrogen gas stream. They were then stored at 4 °C until further use. 

### 2.7. Characterisation of Prepared Solid Stamp Materials 

The surface morphology of bare PDMS and AuNPs–PDMS stamps was analysed using a field emission scanning electron microscope (FE-SEM, J.S.M. 6700F) manufactured by JEOL. Samples for FE-SEM were kept in the dry box before sample preparation for analysis. These samples were attached to a sample holder using carbon tape and then coated with platinum for 90 s using a sputtering coater (JEOL LFC-1300) before the examination. To confirm the presence of AuNPs on the surface of the PDMS stamps, XRD analysis was performed using a Bruker D8 Advance Powder X-ray diffractometer. All solid samples of bare PDMS stamps (Section 2.3), AuNPs–PDMS stamps (Section 2.4) and AuNPs–PDMS stamps with aptamer immobilised on the surface (Section 2.6) were analysed by UV-Vis spectrometer (UV–Vis-NIR, Carry 5000, Varian New South Whales, Australia) in the spectral range of 200 nm–800 nm.

### 2.8. Patterning of Target Protein through Micro-Contact Printing

The areas of the PDMS stamp with immobilised aptamer were covered with the target gp-120 protein solution, followed by incubation at 37 °C for 1 h in a self-made humidified chamber. After incubation, the PDMS stamp was rinsed with RNase-free water and dried under a nitrogen gas stream. The aptamer and gp-120 protein complex immobilised PDMS stamp surface was brought into conformal contact with a UV treated DMOAP-coated slide. To ensure perfect contact weight, three glass slides were placed on top of this assembly during conformal contact. After 10 min of contact in a self-made humidified chamber, the stamp was peeled off from the DMOAP-coated glass slide surface. Then this glass slide surface was dried under a stream of nitrogen gas before LCs analysis.

### 2.9. Fabrication of LCs Optical Cell 

An LCs optical cell was constructed by putting together a sample glass slide and a DMOAP-coated glass slide. At both ends of the two glass slides, mylar films of thickness 6 mm were employed to separate the two glass slides. Then these glass slides were secured with binder clips at both ends. Capillary force was used to fill the area between the two glass slides with around 10 µL of LCs. After 15 min, the constructed LCs optical cell was examined with a 1× objective lens using a transmission mode polarised optical microscope (Nikon ECLIPSE LV100POL, Tokyo, Japan).

### 2.10. Stability Check of the Prepared AuNPs–PDMS Stamp

To check the stability of the prepared PDMS–gold nanoparticles stamp, we used 3′ 6-FAM-conjugated thiolated aptamer (Apt 8). First, a drop of the aptamer solution was dispensed on the surface of the PDMS stamps with and without gold nanoparticles with the help of a micropipette and kept in a self-made humidified chamber at 4 °C for 12 h. After incubation, the stamps were thoroughly rinsed with RNAase-free water to remove any unbound or physically adsorbed aptamer. They were then dried under a nitrogen gas stream. Both PDMS stamps were brought into conformal contact with a DMOAP-coated glass slide separately. After 10 min of contact, the stamp was peeled off from the DMOAP-coated glass slide surface. Then this glass slide surface was dried under a stream of nitrogen gas in preparation for florescence analysis. The fluorescence signals were observed under a fluorescence microscope (Eclipse E200, Nikon, Tokyo, Japan) using a 6 s exposure time.

## 3. Results and Discussion

### 3.1. Proposed Scheme of μCP of Gp-120 Protein Based on AuNPs–PDMS Composite Stamp

**Figure 1:**
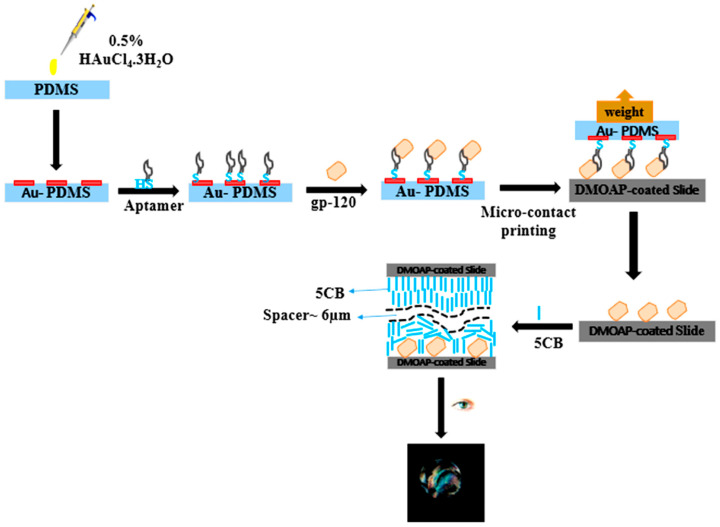
Schematic illustration of the fabrication of AuNPs–PDMS stamp and biorecognition process, including inking with probe aptamer, stamping and patterning target protein gp-120 on DMOAP-coated glass slide through µCP.

In the proposed study, based on gold-thiol chemistry, an appropriate amount of thiol-modified B40t77 aptamer was allowed to immobilize onto the AuNPs–PDMS stamp. After immobilization, the B40t77 aptamer immobilized spot areas were incubated with gp-120 solution before performing μCP. Next, the AuNPs–PDMS stamp with complex was further used for gp-120 protein patterning on the glass slide surface. This patterning step was carried out in a Petri plate by creating handmade humid environment inside the Petri plate; we observed that the humid environment aids in µCP by allowing the protein to fall off from the stamp surface onto the glass slide surface. Finally, a glass slide patterned with target protein was used to fabricate LCs optical cells for imaging protein patterning results in the form of final optical responses of LCs due to their orientational transition. The LCs final optical images analysis showed that the presence of the target protein gp-120 transferred onto the surface of UV-treated DMOAP-coated glass slide leads to disruption of the LCs homeotropic alignment, which resulted in the bright image area. In contrast, the dark image areas were produced due to LCs homeotropic alignment induced by DMOAP (the orientational agent), which is coated on the glass slides surfaces (Figure 1).

### 3.2. Characterisation of AuNPs–PDMS Composite Stamp

**Figure 2:**
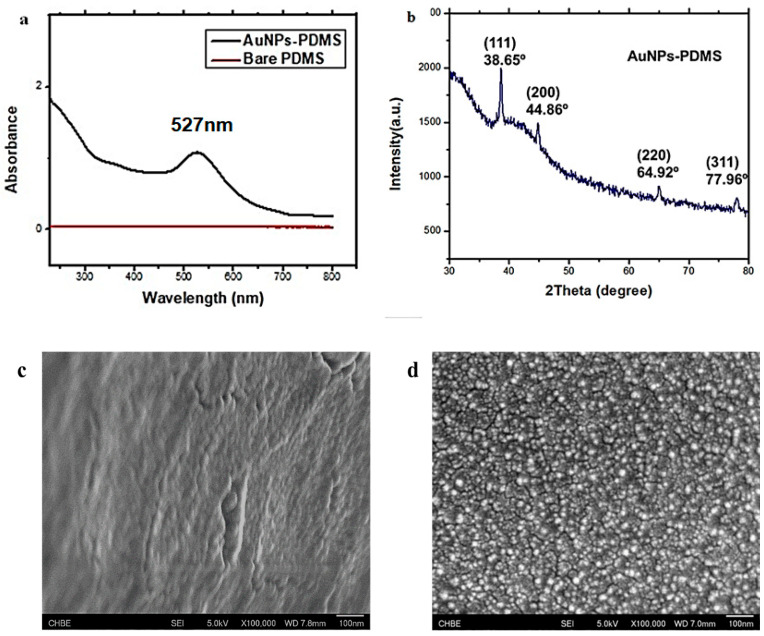
UV-vis absorption spectra. (**a**) UV-Vis spectra of bare PDMS and AuNPs–PDMS, (**b**) XRD of AuNPs–PDMS, (**c**) FeSEM image of bare PDMS and, (**d**) AuNPs–PDMS composite.

There was a visible difference in colour between the prepared bare PDMS stamps and the AuNPs–PDMS stamps. The bare PDMS stamps were transparent, but after surface modification, they turned red, which indicates the presence of AuNPs on the surface of the PDMS. Further, the AuNPs–PDMS stamps were characterized by UV-Vis spectroscopy, X-ray Diffraction (XRD) and Field emission Scanning Electron Microscope (FeSEM). Figure 2a shows UV-Vis spectra of bare PDMS with no absorbance peak, whereas AuNPs–PDMS exhibited an absorbance peak at 527 nm showing the characteristic peak of AuNPs, and the result was consistent with previous studies [12,19]. Figure 2c shows the scanning electron micrograph of bare PDMS and surface modified PDMS with AuNPs, which clearly indicates the presence of AuNPs on the surface of PDMS, as presented in Figure 2d. As can be observed in Figure 2b, the presence of AuNPs on the PDMS stamp surface was confirmed by XRD analysis. The obtained data was also corroborated with the UV-Vis spectroscopy analysis results. The XRD analysis of the AuNPs–PDMS stamp and obtained profiles were confirmed from the JCPDS (04-0784) with a face-centered cubic (F.C.C.) crystal system. XRD peaks at 38.65, 44.86, 64.92 and 77.96, corresponding to 111, 200, 220 and 311 planes, respectively, confirmed the structure. The calculated interplanar spacing distances were 2.3331, 2.0199, 1.4339 and 1.225, respectively, which also corroborated previously reported results [15]. Furthermore, the prepared AuNPs–PDMS stamp also has long-term stability. After six months of storage in a refrigerator, the stamps still exhibited similar visible colour with little performance loss. 

### 3.3. Characterisation of Aptamer Immobilised on PDMS–AuNPs Composite Stamps

**Figure 3:**
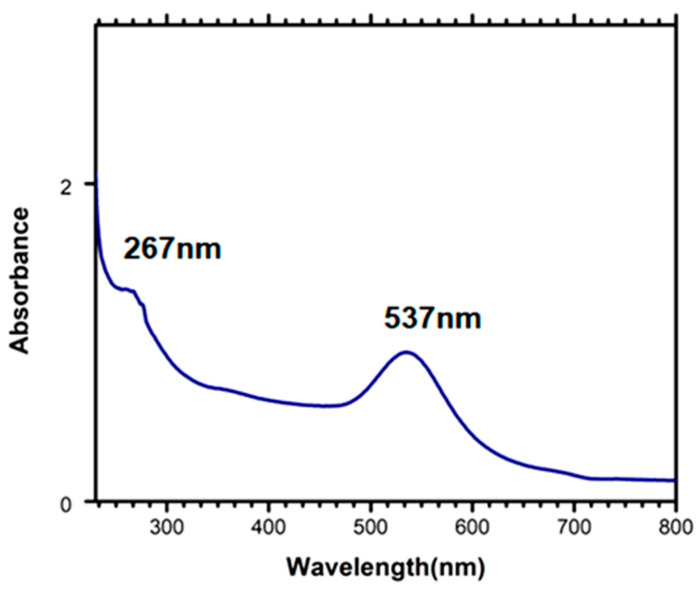
UV-Vis absorption spectra of AuNPs–PDMS with immobilized biorecognition aptamer probe.

Unlike the many previous studies in which bioreceptor probes are randomly attached to the PDMS stamps through electrostatic interactions, we proposed AuNPs–PDMS as a µCP stamp which provides the surface for attachment of thiol-modified biorecognition probes linked to the stamp surface through a dative bond with a single anchoring point based on thiol chemistry. To investigate whether the aptamers were successfully loaded on the surface of the AuNPs–PDMS stamps, UV-Vis absorption spectroscopy analysis was employed (Figure 3). After the attachment of aptamer on the surface of the AuNPs–PDMS stamp, the absorption value increased to 276 nm, indicating the presence of the aptamer, which has a characteristic peak at 260 nm. Hence, these results confirmed the successful attachment of aptamer at the stamp surface. The formation of aptamer bound on AuNPs–PDMS was further confirmed by the red shift in the absorption peak from 527 nm (AuNPs) to 537 nm, along with its increasing absorption intensity.

**Figure 4:**
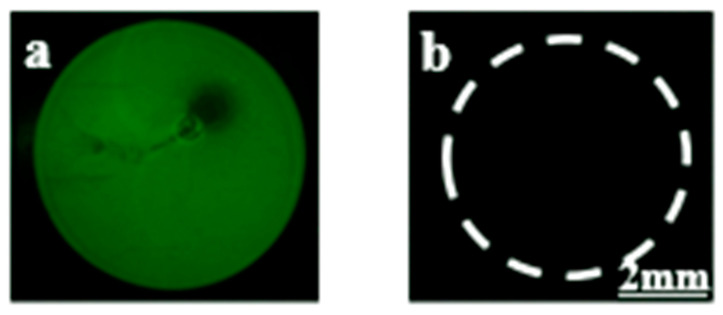
Fluorescence images showing (**a**) AuNPs–PDMS stamp surface with a circular region of immobilised RNA probe (3’ 6-FAM-conjugated Apt 8), and (**b**) DMOAP-coated glass slide surface after µCP. The RNA probe is stable and not transferred from the stamp to the DMOAP-coated glass slide during µCP.

Moreover, we also investigated the effect of AuNPs on the surface of the PDMS stamp on the immobilization of 3′ 6-FAM labelled Apt 8. In this case, we dispensed an Apt 8 solution drop on the AuNPs–PDMS stamp to attach a biorecognition probe on the stamp surface. Afterwards, this stamp was analyzed under a fluorescence microscope. As shown in Figure 4a, a green circular fluorescence signal could be observed, indicating successful attachment of the biorecognition probe (Apt 8) on the AuNPs–PDMS stamp. When µCP was carried out with this stamp, no fluorescence signal was observed, indicating no transfer of biorecognition probe onto the glass slide during µCP (Figure 4). Hence, the biorecognition probe is stable and not transferred from the stamp to the DMOAP-coated glass slide during µCP. These results indicated that AuNPs on the surface of the PDMS stamp play an important role in immobilizing the biorecognition probe on the stamp surface.

### 3.4. Detection of Gp-120 Target Protein through µCP by Using LCs

**Figure 5:**
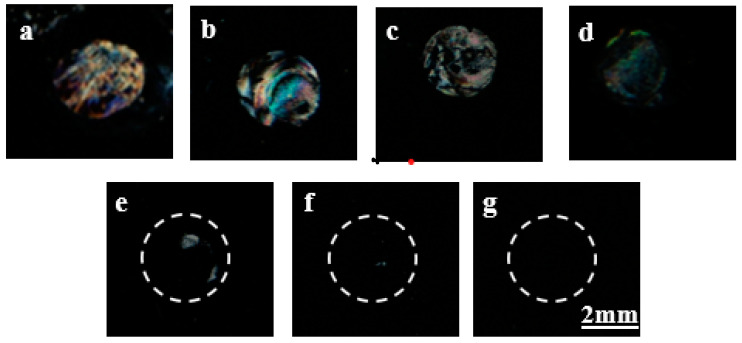
Polarized optical images of LCs supported on DMOAP-coated glass slide printed by AuNPs–PDMS stamp with immobilized probe (B40t77 aptamer) incubated with (**a**) 8 (µg/mL), (**b**) 6 (µg/mL), (**c**) 4 (µg/mL), (**d**) (2 µg/mL), (**e**) 1 (µg/mL), (**f**) 0.5 (µg/mL) and (**g**) (0 µg/mL) of target protein gp-120.

After protein patterning by the AuNPs–PDMS stamp with the immobilized B40t77 aptamer, POM images of the LCs supported on DMOAP-coated slides were obtained and are shown in Figure 5. Before performing µCP, the stamps with attached aptamer were incubated with gp-120 solution. A droplet of a solution containing nonfluorescent gp-120 in different concentrations (8, 4, 2, 1, and 0.5 µg/mL) was placed on the already attached circular area on AuNPs–PDMS with attached B40t77 aptamer. The substrate was kept in a hand-made humid environment in a Petri plate for 1 h at room temperature. A humid environment provides enough time for the probe to interact with its target protein and avoid immediate drying of the solution. After rinsing and drying under a nitrogen stream, the AuNPs–PDMS stamp with the attached B40t77 aptamer and target protein gp-120 complex on its surface was used to print the target protein. Following the procedure, the DMOAP-coated glass slide was exposed to UV to study the transfer of target protein gp-120 during µCP. As previously reported, the UV treatment can strengthen the interactions between the target proteins and glass slides, and protein transfer efficiency strongly depends on interactions between the protein and the surface [7]. This DMOAP-coated glass slide with gp-120 protein printed on its surface was employed for the fabrication of the LCs optical cell for µCP. Bright LCs optical images in Figure 5 show the presence of transferred protein on the slide surface, which disrupt the LCs orientation, indicating successful transfer of the target protein gp-120 from the stamp surface to the DMOAP-coated glass slide. In contrast, the dark areas are due to the absence of the target protein or an insufficient amount of transferred protein which is unable to disrupt the LCs orientation, as in the case of 0 (just 1× PBS), 0.5 and 1 µg/mL concentrations of target protein gp-120 when dispensed on stamps containing the probe aptamer for biorecognition process followed by transferring to slide surface. Previously, John et al., reported an electrochemical aptasensor for HIV-1 gp-120 in which they proposed a dendrimer/streptavidin platform for B40 aptamer attachment and used this aptamer to electrochemically detect gp-120 with a limit of detection of 0.2 nM [31]. Despite of the fact that our proposed limit of detection (LoD) is higher than previously reported, the present biosensing technique is easy to operate, simple and allows detection of gp-120 from the protein mixture. Moreover, compared with our previously reported aptasensors for gp-120 detection [21,23], our present proposed sensing technique not only detects gp-120 from the protein mixture but also transfers the detected gp-120 protein onto a glass substrate for further qualitative analysis. 

### 3.5. Capturing Desired Target Protein (Gp-120) in a Protein Mixture Solution

**Figure 6:**
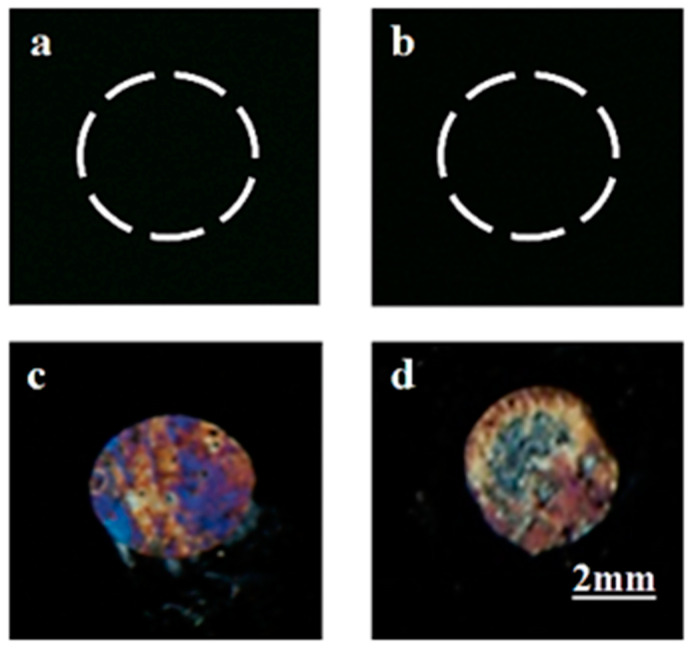
Specificity of the AuNPs–PDMS stamp with immobilized B40t77 aptamer probe after incubation with a solution of the mixture of two proteins (FITC-IgG + gp-120) is demonstrated by fluorescence images with no signals show no binding and transfer of FITC-IgG, (**a**) when 8 µg/mL quantity of gp-120 and FITC-IgG were mixed, and (**b**) when 8 µg/mL of gp-120 and 16 µg/mL of FITC-IgG were mixed. Bright optical images of LCs showing selective binding and transfer of gp-120 onto DMOAP-coated glass slide, (**c**) when 8 µg/mL quantity of gp-120 and FITC-IgG were mixed, and (**d**) when 8 µg/mL of gp-120 and 16 µg/mL of FITC-IgG were mixed.

To test the selectivity of the proposed µCP setup, we further investigated the ability of this µCP stamp with aptamer (B40t77) fixed on its surface to capture the desired target protein gp-120 (nonfluorescent) from a solution containing a mixture of two proteins (target protein + one other protein labelled with a fluorophore). For this test, two solutions of gp-120 with a concentration of 8µg/mL were prepared. The solutions were mixed with FITC labelled IgG with concentrations of 8 and 16 µg/mL. Then these two solutions were incubated with immobilized B40t77 aptamer on the surfaces of two different AuNPs–PDMS stamps. After incubation, the µCP results were analyzed under a polarized optical microscope and a fluorescence microscope. The LCs optical and fluorescence signal images captured are shown in Figure 6. Considering Figure 6a,b, the black fluorescence images with no signal indicates that FITC labelled IgG was not captured and transferred onto the glass slide by the AuNPs–PDMS stamp with B40t77 aptamer immobilized on its surface. For analyzing the transfer of gp-120, the LCs orientational response was exploited to predict the results (Figure 6c,d). These results show that the gp-120 target protein can be captured by the AuNPs–PDMS stamp with attached B40t77 aptamer, even if the gp-120 target protein solution is mixed with FITC labelled IgG solution.

## 4. Conclusions

A simple and cost-effective method of patterning desired protein for detection purposes is proposed in the present investigation. This patterning method captures a specific target protein, even from a mixture containing more than one protein, by a biorecognition probe attached to the stamp surface and then transferred to a desired solid substrate. AuNPs–PDMS stamps were exploited as a suitable stamp surface for attachment of the biorecognition probe. Furthermore, the AuNPs–PDMS stamps were fabricated by the already reported method for in situ modification of PDMS stamps with gold nanoparticles. Using this stamp material, it was found that protein patterning became much more effective by avoiding the possibility of protein degradation and aggregation, which usually occurs in the case of the direct adsorption of proteins on a solid substrate.

Furthermore, it could be seen that the dative bond (Au-SH) between AuNPs from the AuNPS–PDMS stamp surface and the thiol group of the biorecognition probe was strong enough to hold the probe with the stamp surface during µCP. The target protein can be separated from the probe during µCP and successfully transferred to a UV treated DMOAP-coated glass slide. The B40t77 aptamer was used as a biorecognition probe that has specificity for HIV-1 surface glycoprotein gp-120. Therefore, this patterning method allowed detection of gp-120 as the probe selectively binds to gp-120 from the solution and transfers it onto a glass slide. For analyzing the results of µCP, the LC’s orientational response was exploited as a label-free approach for identifying transferred protein. The reported results demonstrate the significant potential of the developed procedure for the fabrication of bio-sensing systems for various analytes.

## Data Availability

Not available from the authors.

## References

[1] Sun H., Chen G.Y.J., Yao S.Q. (2013). Review Recent Advances in Microarray Technologies for Proteomics. Chem. Biol..

[2] Hölz K., Schaudy E., Lietard J., Somoza M.M. (2019). Multi-level patterning nucleic acid photolithography. Nat. Commun..

[3] Ogaki R., Alexander M., Kingshott P. (2010). Chemical patterning in biointerface science Patterning of surfaces with different chemistries provides novel insights of new chemically patterned surfaces is highlighted. Mater. Today.

[4] Delamarche E., Pereiro I., Kashyap A., Kaigala G.V. (2021). Biopatterning: The Art of Patterning Biomolecules on Surfaces. Langmuir.

[5] Juste-Dolz A., Avella-Oliver M., Puchades R., Maquieira A. (2018). Indirect microcontact printing to create functional patterns of physisorbed antibodies. Sensors.

[6] Bernard A., Renault J.P., Michel B., Bosshard H.R., Delamarche E. (2000). Microcontact printing of proteins. Adv. Mater..

[7] Chen C.H., Yang K.L. (2011). Improving protein transfer efficiency and selectivity in affinity contact printing by using UV-modified surfaces. Langmuir.

[8] Miranda I., Souza A., Sousa P., Ribeiro J., Castanheira E.M.S., Lima R., Minas G. (2022). Properties and applications of PDMS for biomedical engineering: A review. J. Funct. Biomater..

[9] Gökaltun A., Kang Y.B., Yarmush M.L., Usta O.B., Asatekin A. (2019). Simple Surface Modification of Poly(dimethylsiloxane) via Surface Segregating Smart Polymers for Biomicrofluidics. Sci. Rep..

[10] Tu Q., Wang J.C., Zhang Y., Liu R., Liu W., Ren L., Shen S., Xu J., Zhao L., Wang J. (2012). Surface modification of poly(dimethylsiloxane) and its applications in microfluidics-based biological analysis. Rev. Anal. Chem..

[11] Wang W., Wu W.Y., Zhong X., Wang W., Miao Q., Zhu J.J. (2011). Aptamer-based PDMS-gold nanoparticle composite as a platform for visual detection of biomolecules with silver enhancement. Biosens. Bioelectron..

[12] Zhang Q., Xu J.J., Liu Y., Chen H.Y. (2008). In-situ synthesis of poly(dimethylsiloxane)-gold nanoparticles composite films and its application in microfluidic systems. Lab Chip.

[13] Wang Q., Zhou J., Wu X., Riaud A. (2021). Optimization of synthesis conditions of gold nanoparticlespolydimethylsiloxane composite for ultrasound generation. Nanotechnology.

[14] Zhu A., Ali S., Xu Y., Ouyang Q., Chen Q. (2021). A SERS aptasensor based on AuNPs functionalized PDMS film for selective and sensitive detection of Staphylococcus aureus. Biosens. Bioelectron..

[15] Yao J.Y., Fostier A.H., Santos E.B. (2020). In situ formation of gold and silver nanoparticles on uniform PDMS films and colorimetric analysis of their plasmonic color. Colloids Surfaces A Physicochem. Eng. Asp..

[16] Pusty M., Shirage P.M. (2020). Gold nanoparticle-cellulose/PDMS nanocomposite: A flexible dielectric material for harvesting mechanical energy. RSC Adv..

[17] SadAbadi H., Badilescu S., Packirisamy M., Wüthrich R. (2013). Integration of gold nanoparticles in PDMS microfluidics for lab-on-a-chip plasmonic biosensing of growth hormones. Biosens. Bioelectron..

[18] Xue Y., Li X., Li H., Zhang W. (2014). Quantifying thiol-gold interactions towards the efficient strength control. Nat. Commun..

[19] Wu W.Y., Bian Z.P., Wang W., Wang W., Zhu J.J. (2010). PDMS gold nanoparticle composite film-based silver enhanced colorimetric detection of cardiac troponin I. Sens. Actuators B Chem..

[20] Li Y., Liu S., Ling L., Xue C.Y., Chin S.Y., Khan S.A., Yang K.L., Tu Q., Wang J.C.J., Zhang Y. (2018). Recent developments in aptasensors for diagnostic applications. Langmuir.

[21] Abbasi A.D., Hussain Z., Yang K.L. (2021). Aptamer laden liquid crystals biosensing platform for the detection of HIV-1 glycoprotein-120. Molecules.

[22] Wang Q., Yang Q., Wu W. (2020). Graphene-Based Steganographic Aptasensor for Information Computing and Monitoring Toxins of Biofilm in Food. Front. Microbiol..

[23] Abbasi A.D., Hussain Z., Liaqat U., Arif D., Yang K.-L. (2021). Liquid Crystal Based Binding Assay for Detecting HIV-1 Surface Glycoprotein. Front. Chem..

[24] Dey A.K., Griffiths C., Lea S.M., James W. (2005). Structural characterization of an anti-gp120 RNA aptamer that neutralizes R5 strains of HIV-1. RNA.

[25] Chen J., Liu Z., Yang R., Liu M., Yao J., Zhang M., Li N., Yuan Z., Jin M., Shui L. (2022). A label-free optical immunoassay based on birefringence of liquid crystal for insulin-like growth factor-I sensing. Sens. Actuators B Chem..

[26] Lin C.T., Hsu W.T., Hwang S.J. (2021). Real-time liquid crystal-based creatinine sensor using a micro-patterned flexible substrate. Liq. Cryst..

[27] Khan M., Liu S., Qi L., Ma C., Munir S., Yu L., Hu Q. (2021). Liquid crystal-based sensors for the detection of biomarkers at the aqueous/LC interface. TrAC Trends Anal. Chem..

[28] Hong P.T.K., Jang C.H. (2021). Simple, sensitive technique for α-amylase detection facilitated by liquid crystal-based microcapillary sensors. Microchem. J..

[29] Yin F., Cheng S., Liu S., Ma C., Wang L., Zhao R., Lin J.M., Hu Q. (2021). A portable digital optical kanamycin sensor developed by surface-anchored liquid crystal droplets. J. Hazard. Mater..

[30] SadAbadi H., Badilescu S., Packirisamy M., Wuẗhrich R. (2012). PDMS-gold nanocomposite platforms with enhanced sensing properties. J. Biomed. Nanotechnol..

[31] John S.V., Khati M., Mamba B.B., Arotiba O., Rotherham L.S. (2014). Towards HIV detection: Novel Poly (propylene) dendrimer-streptavidin platform for electrochemical DNA and gp-120 aptamer biosensors. Int. J. Electrochem. Sci..

